# Cobalt Administration Causes Reduced Contractility with Parallel Increases in TRPC6 and TRPM7 Transporter Protein Expression in Adult Rat Hearts

**DOI:** 10.1007/s12012-018-9498-3

**Published:** 2018-12-06

**Authors:** Sarunya Laovitthayanggoon, Catherine J. Henderson, Claire McCluskey, Margaret MacDonald, Rothwelle J. Tate, M. Helen Grant, Susan Currie

**Affiliations:** 1 Department of Biomedical Engineering, University of Strathclyde, Glasgow, G4 0RE Scotland UK; 2 Strathclyde Institute of Pharmacy and Biomedical Sciences, University of Strathclyde, Glasgow, G4 0RE Scotland UK

**Keywords:** Cardiotoxicity, Cobalt, Cardiac fibroblast, Transient receptor potential (TRP) channels, Divalent metal transporter channel 1 (DMT1)

## Abstract

Exposure to circulating cobalt (Co^2+^) in patients with metal-on-metal orthopaedic hip implants has been linked to cardiotoxicity but the underlying mechanism(s) remain undefined. The aim of the current study was to examine the effects of Co^2+^ on the heart in vivo and specifically on cardiac fibroblasts in vitro. Adult male rats were treated with CoCl_2_ (1 mg/kg) for either 7 days or 28 days. Inductively coupled plasma mass spectrometry (ICP-MS) was used to measure Co^2+^ uptake into various organs of the body. Co^2+^ accumulated in the heart over time with significant levels evident after only 7 days of treatment. There was no evidence of cardiac remodelling following Co^2+^ treatment as assessed by heart weight:body weight and left ventricular weight:body weight. However, a decrease in fractional shortening, as measured using echocardiography, was observed after 28 days of Co^2+^ treatment. This was accompanied by increased protein expression of the ion transient receptor potential (TRP) channels TRPC6 and TRPM7 as assessed by quantitative immunoblotting of whole cardiac homogenates. Uptake of Co^2+^ specifically into rat cardiac fibroblasts was measured over 72 h and was shown to dramatically increase with increasing concentrations of applied CoCl_2_. Expression levels of TRPC6 and TRPM7 proteins were both significantly elevated in these cells following Co^2+^ treatment. In conclusion, Co^2+^ rapidly accumulates to significant levels in the heart causing compromised contractility in the absence of any overt cardiac remodelling. TRPC6 and TRPM7 expression levels are significantly altered in the heart following Co^2+^ treatment and this may contribute to the Co^2+^-induced cardiotoxicity observed over time.

## Introduction

Cardiotoxicity is the most frequently encountered adverse effect contributing to attrition of new candidate pharmaceuticals, but it has not often been associated with use of medical devices. Hip joint replacements are one of the most successful elective surgeries, but they are not without their limitations. The most notorious of these limitations in recent years are the adverse effects associated with metal-on-metal (MoM) hip articulations, made of CoCr alloy, which have led to several implants being removed from use following release of warning alerts by the medicines and healthcare products regulatory agency (MHRA) [1]. Wear of these medical devices causes formation and release of nanoparticulate debris of CoCr, and metal ions in patients. Both Co and Cr ions are released into the bloodstream. Co ions, being more soluble than Cr ions, enter the patients’ circulation and may evoke systemic adverse effects including cardiomyopathy, CNS toxicity and hypothyroidism in the patients over time. Use of metal-on-metal articulations in hip replacement was at its height in 2006, but has now declined to contribute < 1.1% of total hip replacements implanted. Although most of the defective MoM designs have been removed from the market through the actions of MHRA, there are still over 10,000 patients in the UK alone with these implants still in situ. If an artificial hip joint fails, revision surgery is carried out to replace it, but this can involve considerable morbidity risk for the patient. Currently, litigation against the manufacturers is in progress in many countries, led by the USA, and it is important to determine what controls susceptibility to adverse effects, and how to limit further damage to the health of these patients.

Cardiotoxicity was first associated with Co^2+^ in the 1960s when beer drinkers in Canada showed symptoms of cardiomyopathy after Co^2+^ had been used as an antifoaming agent during the brewing process [2]. In some of the beer drinkers, malnutrition may have contributed to susceptibility to the effects of Co^2+^, but several cases of cardiomyopathy have also been reported in metal workers [3, 4] and in patients with MoM implants in situ [5, 6]. Studies using echocardiography have described altered diastole, severely reduced left ventricular systolic function and cardiac hypertrophy [7]. It has also been shown that Co^2+^ accumulates in the hearts and other organs of animals treated with Co^2+^ and also with CrCo alloy nanoparticles [8, 9]. The underlying mechanisms for Co^2+^ transport into the cells of the heart remain unclear.

There are a large number of proteins dedicated to transporting calcium (Ca^2+^) and magnesium (Mg^2+^) ions into cells, and across cellular compartments. These vital ions are versatile second messengers controlling signalling pathways which mediate many physiological and pathological processes. Co^2+^ is also a divalent cation, and as such may enter cells by mimicking Ca^2+^ and by using similar transport routes. TRP proteins belong to the TRP superfamily of Ca^2+^ permeable channels exhibiting diverse tissue distribution, subcellular localisation, and physiological function and may play a role in Co^2+^ uptake. Mammalian TRP channels can be divided into various subfamilies that include TRP canonical (TRPC1-6), TRP vanilloid (TRPV1-6) and TRP melastatin (TRPM1-8). Most of the TRP channels are located in the plasma membrane and function as the driving force for Ca^2+^ and Mg^2+^ transport [10]. Interestingly, some studies have indicated that Co^2+^ may alter expression of TRP channels and be transported by these channels. Particular emphasis has been on TRPC6 and TRPM7 [11, 12]. Pertinent to the current study is the fact that TRP channels are highly expressed in the heart and have been widely studied in cardiomyopathy. High expression of TRPC1, TRPC3 and TRPC6 genes are seen in heart disease and contribute to remodelling of the heart [13]. These channels are expressed across both contractile and non-contractile cells of the heart and directly influence physiological and pathological responses of both [14]. It therefore seems possible that TRP channels may also be directly involved in cardiotoxic responses, such as those induced by Co^2+^. Another transporter involved in the transport of divalent cations is divalent metal ion transporter 1 (DMT1). This protein is known to transport iron and a range of other cations via a proton-coupled mechanism and is expressed in heart [15]. Co ions are predicted to enter brain cells via this transporter [16] and DMT1 in neurones has been shown to exhibit altered functional characteristics following exposure to Co^2+^ [17]. Although nothing is known of whether DMT1 may be involved in Co^2+^ uptake into the heart, evidence for involvement across other systems does make this a possibility.

Our hypothesis is that Co^2+^ treatment impairs cardiac function in vivo and this impairment is reflected at a cellular level where alterations in transporter proteins are evident. Experiments were designed to find out if Co^2+^ is cardiotoxic to animals dosed at concentrations that mimic the blood concentrations measured in MoM patients. Effects of Co^2+^ on primary adult rat cardiac fibroblasts (CFs) were also investigated since these cells are responsible not only for maintaining the structural integrity of the heart, but also for modulating electrical coupling between neighbouring myocytes [18]. A key advantage of focusing on adult CFs instead of the contractile myocytes is that the CFs can be maintained in culture over several passages, enabling longer-term investigation of Co^2+^ effects. This is not possible with adult cardiac myocytes that lose their characteristic adult phenotype very quickly in culture. Specifically, we have studied the uptake characteristics of Co^2+^ into the organs of the body as well as into primary isolated CFs. Candidate proteins (TRPC6, TRPM7 and DMT1) that may regulate the uptake of Co^2+^ into the heart have been examined. Parallel alterations in expression of each of these proteins were observed across whole cardiac tissue homogenates and CFs following Co^2+^ treatment. This could highlight a role for these proteins in mediating the effects of Co^2+^ in the heart.

## Methods

### In Vivo Cobalt Treatment and Echocardiography

Male Sprague–Dawley rats [200–300 g (8–10 weeks old)] were given intra-peritoneal (i.p.) injections of distilled water (*n* = 3) or 1 mg/kg CoCl_2_ (*n* = 6) in distilled water daily for 7 days and 28 days. The animals were subjected to echocardiography (short-axis view) as previously described [19] at both 7 days and 28 days and then euthanised on the same day as the final injection. Procedures complied with the ARRIVE guidelines and conformed to the Guide for the Care and Use of Laboratory Animals published by the US National Institutes of Health (NIH Publication No. 85-23, revised 1996) and Directive 2010/63/EU of the European Parliament. Blood was collected by cardiac puncture and specific organs (liver, brain, spleen, heart, lungs, kidneys and testes) were removed, weighed and then collected for further analysis by ICP-MS.

Body weights were recorded daily (in the morning). Recording body weight during Co^2+^ treatment provides an indicator of the general health status of the rats that may be relevant in the interpretation of toxic effects.

### ICP-MS Analysis

Blood (0.5 mL), organ samples (100 mg) or cells (confluent 35 mm^2^ dish) were digested with 0.5 mL of HNO_3_ [70% (v/v)] and heated at 103 °C for 20 min. Hydrogen peroxide [H_2_O_2_, 30% (w/v)] (0.25 mL) was added and the digestion continued for a further 20 min. Samples were stored at − 20 °C prior to metal analysis. For analysis, samples were thawed and diluted 40-fold (cell lysates tenfold) in ultrapure water containing 1% (v/v) HNO_3_. Co^2+^ standards (range 0, 50, 100, 250, 500 and 1000 µg/L) were prepared by diluting 1000 mg/L CoCl_2_ (TraceCERT®) with HNO_3_. Samples and standards were analysed using an Agilent 7700× octopole collision system ICP-MS (Agilent Technologies; Wokingham, UK) in helium gas mode using Scandium (Sc, 3 ppm) as an internal standard. The quantification was based on the maximum signal for a particular isotope, also referred to as peak height. The limit of detection for Co^2+^ is 0.3 ng/L and the limit of quantification is 1 ng/L. Five readings were taken, and the result obtained is the mean value.

### Cardiac Fibroblast Isolation, Culture and Co^2+^ Treatment

CFs were isolated by bulk digestion using collagenase and protease as previously described [20]. Cells were cultured at 37 °C and 5% CO_2_ in Dulbecco’s Modified Essential Medium (DMEM) containing 20% foetal calf serum (FCS) and were passaged at ~ 80% confluence. CFs were only used up to passage 3 as we noted a myofibroblast phenotype starting to emerge beyond this point. For treatment with Co^2+^ and further analysis by immunoblotting, cells were seeded into T25 flasks at a density of 1 × 10^5^ cells/mL. After 24 h, CoCl_2_ (10 µM) was added and cells were left for either 48 h or 72 h prior to stopping the treatment. At the end-point, culture medium was discarded and cells were washed in PBS. Homogenisation buffer (300 µL) (0.1 M Na_2_PO_4_, pH 7.6, 500 µM 4-(2-aminoethyl) benzenesulfonyl fluoride hydrochloride (AEBSF), 150 nM Aprotinin, 1 µM E−64 and 1 µM leupeptin) was added, cells scraped from the flasks and homogenised using seven strokes of a motor-driven Teflon-glass homogeniser. Homogenates were aliquoted and stored at − 80 °C with one aliquot reserved for total protein quantification.

### Measurement of Total Protein Content

Total protein was measured using a Lowry assay [21] with bovine serum albumin (BSA) used at a range of concentrations (0–200 µg/mL) to generate a standard curve.

### Immunofluorescence

CFs and colonic vascular smooth muscle cells (rat primary cells, used as a positive control) were grown on coverslips until confluent and fixed in 4% (v/v) paraformaldehyde. Cells were exposed to cold methanol then washed in PBS and permeabilised with Triton X-100 [0.01% (v/v)] for 10 min. Non-specific binding was blocked using 1% (w/v) bovine serum albumin (BSA) followed by application of primary antibody [vimentin (abcam #Vim3B4) and α-smooth muscle actin (Sigma-Aldrich #A5228)] overnight at 4 °C. Anti-mouse IgG-FITC (Sigma-Aldrich#FO257) (1:1000) was then applied and coverslips mounted on to slides using Mowiol® (Sigma-Aldrich) mounting medium containing 4′,6-diamidino-2-phenylindole (DAPI) (Vecta laboratory). The DAPI counter-stain in the mounting medium stained the cell nuclei blue.

### MTT (3-(4,5-Dimethylthiazol-2-yl)-2,5-Diphenyltetrazolium Bromide) Assay

CFs were plated in a 96-well plate at 10^4^ cells/cm^2^ and a range of CoCl_2_ concentrations [0–25 µM (low concentration range) or 0–1000 µM (high concentration range)] added. Incubations were performed for 24, 48 and 72 h. MTT was added for 4 h and absorbance measured at 540 nm.

### Quantitative Immunoblotting

Samples (homogenised cardiac tissue or CFs) were analysed by immunoblotting using the NuPAGE system as previously described [19, 20]. DMT1 protein was detected using an overnight incubation in anti-DMT1 (rabbit polyclonal, Sigma-Aldrich) at 1:800 dilution. TRPM7 and TRPC6 proteins were detected using anti-TRPM7 (rabbit polyclonal, Abcam) at 1:500 dilution and TRPC6 (rabbit polyclonal, Sigma–Aldrich) at 1:500 dilution, respectively. GAPDH protein was detected as a loading control (mouse polyclonal, Abcam). All membranes were then subjected to a 2-h incubation in peroxidase-conjugated anti-rabbit or anti-mouse IgG prior to incubation in chemiluminescence reagent and exposure onto X-ray film. Protein bands were quantified using a GS-800 imaging densitometer and Quantity One software (BioRad).

### Statistical Analysis

Results are expressed as mean values ± S.E.M. of *n* observations, where *n* represents the number of samples. Comparisons were assessed using the student’s *t*-test or one-way ANOVA with Dunnett’s post-test as indicated. Differences were considered statistically significant when *p* < 0.05. Where no *p* value is shown, *p* > 0.05.

## Results

### Effects of Co^2+^ Treatment on Body Weight and Individual Organ Weight

**Fig. 1:**
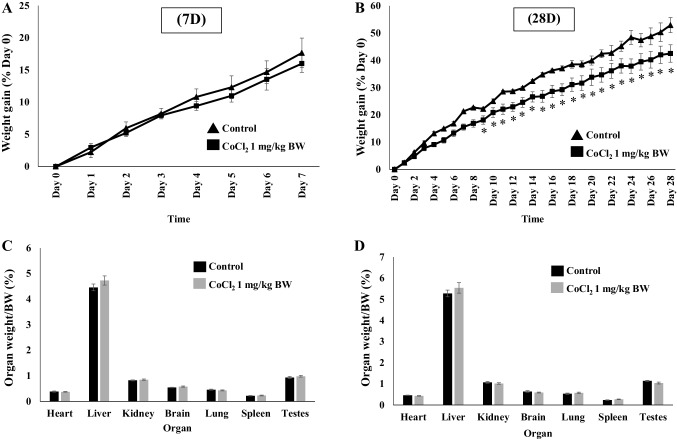
The effect of Co^2+^ treatment on body weight and organ weight. Adult male rats were given daily i.p. injections of 1 mg/kg CoCl_2_ or distilled water (control) for either 7 days or 28 days. Triangles in **a**, **b** represent the body weight gain of rats in control groups (*n* = 3) and squares represent the body weight gain of rats in the Co treated group (*n* = 6). Data are presented as the % weight gain over time compared to day 0. **c**, **d** show histograms representing the organ weights from rats in the control group (*n* = 3) and Co-treated group (*n* = 6) at 7 days (**c**) and 28 days (**d**). Data are presented as the percentage of organ weight/body weight. All data are presented as mean ± SEM, (two-sample *t*-test, *p < *0.05* with respect to control)

Adult male rats were treated with 1 mg/kg CoCl_2_ as described in “Methods” section. Body weight was measured on each day of treatment and recorded. Data show that body weight increased over the period of treatment and the % body weight gain (calculated as a percentage of the initial body weight on day 0) is shown over 7 days and 28 days (Fig. 1a, b). After 7 days of treatment, there was no difference between control and Co^2+^-treated animals, however, from day 8 onwards there were significant differences between the groups. In addition to checking body weights, the weights of individual organs were also recorded from each animal. This is an important consideration for toxicology studies since significant differences in organ weights between control and treatment groups might occur in the absence of any morphological changes. Organ weight data are shown as the ratio of organ weight:body weight to account for any differences in body weight between animals (Fig. 1c, d). No significant differences were apparent in any of the organs measured.

### Cobalt Uptake into Individual Organs

**Fig. 2:**
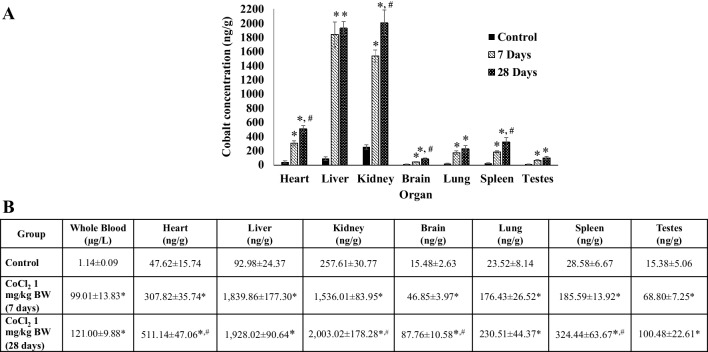
Comparison of Co^2+^ content across different organs following CoCl_2_ treatment. **a** Histogram showing Co^2+^ content in individual organs at day 0 (control), 7 days and 28 days following daily i.p. injections of 1 mg/kg CoCl_2_. **b** Mean values from ICP-MS analysis in µg/L (whole blood) or ng/g (tissue) for Co^2+^ found in each sample set. Control (*n* = 3) and Co^2+^-treated groups (*n* = 6) were reported as mean values ± SEM, (one-way ANOVA,*p < 0.05 with respect to control and, ^*#*^*p* < 0.05 comparing 7 and 28 days)

The distribution of Co ions into the organs and blood of Co^2+^-treated rats following either 7 days or 28 days of treatment was investigated using ICP-MS and measured as ng/g (Fig. 2). Co^2+^ was found to enter all the organs of rats over the period of treatment and there were significant differences in organ content of Co^2+^ between treated animals and those that had no metal ion treatment. Significant accumulation of Co^2+^ in heart, kidney, brain and spleen occurred over 7 days and levels continued to increase up until 28 days. The two organs that accumulated most Co^2+^ were liver and kidney (~ 2000 ng/g) and the Co^2+^ content was 4- to 40-fold higher than that seen in other organs. The Co^2+^ concentration in whole blood taken from Co^2+^-treated rats was almost 100-fold more than blood from untreated rats. The hearts of Co^2+^-treated rats had ~ sixfold greater levels of Co^2+^ after 7 days of treatment and these levels almost doubled (~ 11-fold) following 28 days of treatment.

### Effects of Co^2+^ on In Vivo Cardiac Contractile Performance

**Fig. 3:**
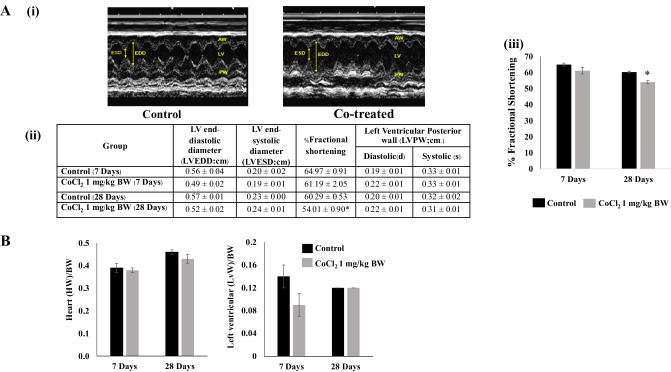
Chronic Co^2+^ treatment reduces fractional shortening in the absence of cardiac remodelling in vivo. **a** (i) Echocardiogram showing M-mode traces in the parasternal short-axis view of the left ventricle (LV) performed in control rats (*n* = 3) and Co^2+^ treated rats (*n* = 6) as indicated. *ESD* end-systolic diameter, *EDD* end-diastolic diameter, *AW* anterior wall, *PW* posterior wall. **a** (ii) Table comparing the echocardiographic parameters of ventricular performance between control and Co^2+^-treated groups. **a** (iii) Histogram comparing fractional shortening between control and Co^2+^-treated groups at 7 days and 28 days. **b** Represents the heart weight and left ventricular weight of rats in the control group and Co^2+^-treated group. Data are presented as the ratio of heart weight and left ventricular weight to body weight ± SEM, (two-sample *t*-test, *p < *0.05* with respect to control)

Contractile performance of the hearts of Co^2+^-treated (*n* = 6) and untreated (*n* = 3) animals from both the 7 days of group and the 28 days of group was monitored by echocardiography and % fractional shortening (%FS) calculated. Typical motion-mode traces taken from the 28 days of group are shown in Fig. 3a (i). Left ventricular end-diastolic diameter (LVEDD) and left ventricular end-systolic diameter (LVESD) were measured and used to calculate %FS. No differences were apparent following 7 days of Co^2+^ treatment, however, after 28 days there was a small but significant reduction in %FS in the Co^2+^-treated group (Fig. 3a (ii) and (iii)). Following echocardiography, whole heart wet weights as well as left ventricular wet weights were measured post-mortem and heart weight:body weight as well as LV weight:body weight ratios calculated. There was no difference in the ratios obtained between untreated and Co^2+^-treated groups at either 7 days or 28 days, indicating no evidence for hypertrophic remodelling of the heart (Fig. 3b).

### Co^2+^-Induced Alterations in TRP Channels in Whole Heart

**Fig. 4:**
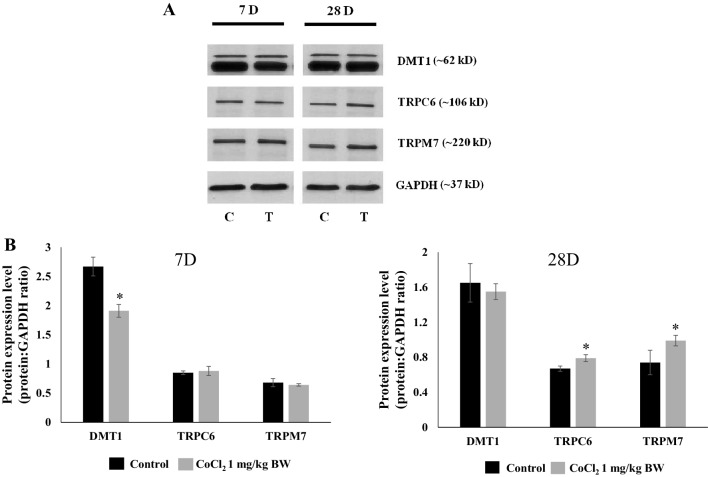
Chronic Co^2+^ treatment increases TRP channel expression in the heart. **a** Representative immunoblots of whole cardiac left ventricular homogenates (20 µg) from control and 7-day or 28-day Co^2+^-treated animals. Blots were probed for either DMT1, TRPC6 and TRPM7 and protein expression quantified using GAPDH as an internal control. **b** Histograms showing mean data presented as ratios of DMT1:GAPDH, TRPC6:GAPDH, or TRPM7:GAPDH from control (*n* = 3) and Co^2+^-treated (*n* = 6) groups following 7 days and 28 days of treatment. Data are presented as means ± S.E.M. and statistical analysis performed using two-sample *t*-tests (*n* ≥ 3, *p < *0.05*, treatment group vs. control group)

To identify possible mechanisms for Co^2+^ uptake into the heart and to test whether chronic Co^2+^ treatment might have the potential to alter these mechanisms, the expression of several ion transporter proteins was examined. Whole left ventricular cardiac homogenates (WH) were prepared from untreated rat hearts and from the hearts of rats treated with CoCl_2_. WHs from rats that had been treated for either 7 days or 28 days were assessed for expression levels of DMT1, TRPC6 and TRPM7 proteins using quantitative immunoblotting. Equivalent total protein loads (20 µg) were analysed and expression compared between control and treated samples for each individual protein (DMT1, TRPC6 and TRPM7). DMT1 was expressed at significantly higher levels in rat heart than either of the TRP channels. Glyceraldehyde 3-phosphate dehydrogenase (GAPDH) was also detected and used as an internal control (Fig. 4a). To enable quantitative comparison, ratios were calculated for DMT1:GAPDH, TRPC6:GAPDH and TRPM7:GAPDH for WHs from untreated rats and Co^2+^-treated rats at 7 days and 28 days (Fig. 4b). Following 7 days of treatment there was a significant drop in DMT1 protein expression in preparations from Co^2+^-treated rats with no apparent changes in TRP channel expression. However, after 28 days of treatment, both TRPC6 and TRPM7 showed increased levels of expression with no changes in DMT1.

### Effects of Co^2+^ on the Viability of Cardiac Fibroblasts

**Fig. 5:**
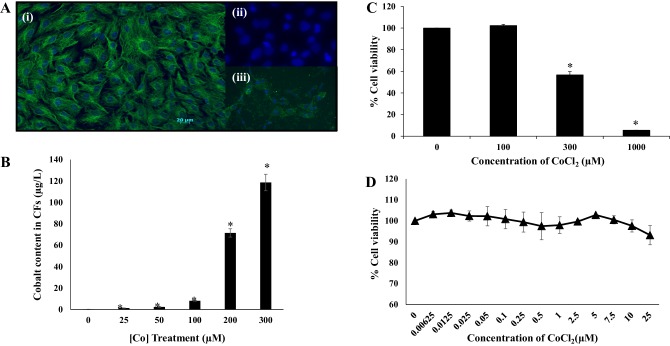
Co^2+^ is transported into cardiac fibroblasts and reduces cell viability. **a** CFs stained with (i) vimentin and DAPI and (ii) α-smooth muscle action and DAPI. (iii) Rat vascular smooth muscle cells stained with α-smooth muscle actin and DAPI. **b** Histogram showing ICP-MS analysis of intracellular Co^2+^ levels in CFs exposed to increasing concentrations of CoCl_2_ (0–300 µM) for 72 h. **c** Histogram showing % cell viability analysed by an MTT assay following exposure of CFs to high concentrations (0–1000 µM) of CoCl_2_ for 72 h and **d** a line graph showing % cell viability as assessed by MTT assay following exposure to a range of low concentrations (0–25 µM) of CoCl_2_ for 72 h. Results are expressed as mean values ± SEM. Statistical analysis was carried out using one-way-ANOVA with post hoc Dunnett’s comparison (*n* = 3, *p < *0.05*)

In order to understand more about where in the heart TRPC6 and TRPM7 channels may be of importance in mediating the effects of Co^2+^, specific examination of rat CFs was performed. These non-contractile cells play an essential role in maintenance of cardiac extracellular matrix and structure as well as influencing cardiac myocyte function. The substantial plasticity of these cells is still under investigation but evidence strongly suggests that CFs play a critical role in cardiac pathophysiology [22]. Co^2+^ uptake into adult rat CFs over 72 h was measured using ICP-MS. Initially, we identified these cells based on vimentin staining and lack of smooth muscle actin staining (Fig. 5a). For uptake into the cells, a range of CoCl_2_ concentrations was assessed (0–300 µM) and there was significant uptake into CFs at concentrations > 100 µM (Fig. 5b). The level of intracellular Co^2+^ following treatment with 25 µM CoCl_2_ was 1.36 ± 0.15 µg/L rising to 8.09 ± 0.4 µg/L with 100 µM CoCl_2_. Following treatment with 300 µM CoCl_2_, intracellular Co^2+^ levels increased to 118.66 ± 7.7 µg/L. As Co^2+^ uptake into cells increased with increasing concentrations of extracellular CoCl_2_, there was a concentration-dependent effect on cell viability with concentrations > 100 µM resulting in reduced viability after 72 h as assessed by MTT assays (Fig. 5c). In order to quantify expression of potential Co^2+^ transporter proteins in CFs following exposure to CoCl_2_, we wanted to ensure that cell viability was not affected. Therefore, we chose to examine the effects of a lower concentration of CoCl_2_ (10 µM) on protein expression. This concentration has no effect on CF viability as assessed by MTT assay following 72-h treatment (Fig. 5d).

### Co^2+^-Induced Alterations in TRP Channels in Cardiac Fibroblasts

**Fig. 6:**
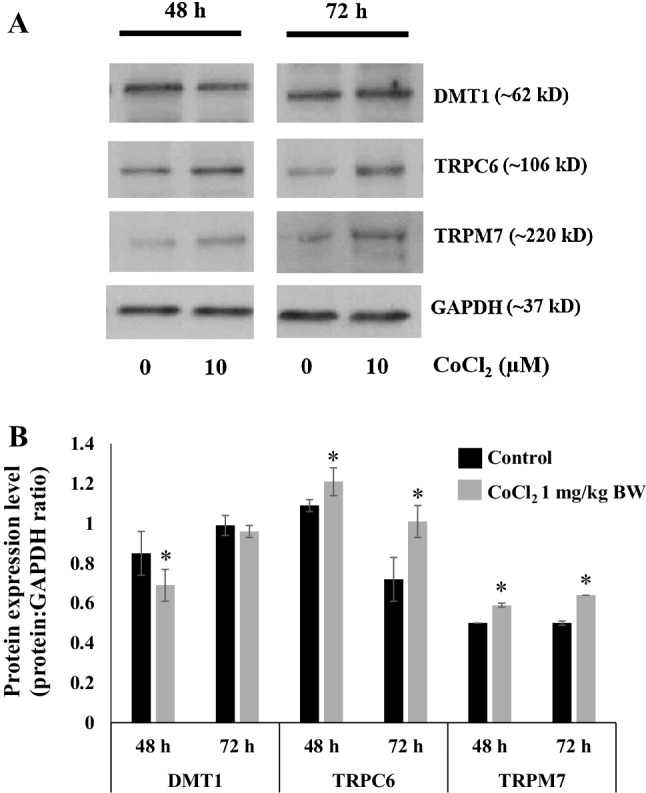
Chronic Co^2+^ treatment of cardiac fibroblasts causes increased expression of TRPC6 and TRPM7. **a** Cells were exposed to CoCl_2_ (10 µM) for 48 and 72 h and total cell extracts (50 µg loads/lane) analysed by immunoblot for DMT1, TRPC6 and TRPM7. GAPDH was detected and used as an internal control. **b** Histogram showing expression of each protein expressed as a ratio to the internal control (GAPDH) under control conditions and following treatment with CoCl_2_. Data are presented as mean values ± SEM. Statistical analysis was carried out using a two-sample *t*-test (*n* ≥ 3, *p < *0.05*, treatment group vs. control group)

CFs were treated with 10 µM CoCl_2_ for up to 72 h and the effect on DMT1, TRPC6 and TRPM7 protein expression assessed by quantitative immunoblotting. All three proteins were expressed in CFs with DMT1 expressed at higher levels that either TRPC6 or TRPM7. After 48-h treatment, DMT1 levels were significantly decreased with significant increases in the levels of both TRP channels. At 72 h, there was no obvious effect of Co^2+^ on DMT1 expression; however, both TRPC6 and TRPM7 proteins were again significantly increased in the presence of CoCl_2_ (Fig. 6a, b).

## Discussion

To our knowledge, this is the first time that Co^2+^-induced events in the whole heart have been linked with effects that are induced in the non-contractile cells of the heart. Co^2+^ is rapidly taken up and distributed throughout all the main organs of the body with significant levels found in the heart after just 7 days of treatment (Fig. 2). Although there are no obvious signs of organ remodelling or hypertrophy over the 28 days of treatment period, the animals receiving CoCl_2_ do show a significant reduction in weight gain after the first week of treatment (Fig. 1). This effect has been reported previously in other species and it has been suggested that Co^2+^ can induce as much as 20–30% weight loss over a period of 10 weeks in dogs, albeit at higher concentrations than used in the current study [23]. This could be due to actions of Co^2+^ on the CNS to regulate appetite [24]. Cardiac function is altered after 28 days of treatment with CoCl_2_ (1 mg/kg) whereby % fractional shortening is significantly reduced (Fig. 3a). The dose of CoCl_2_ used in the current study is very low when compared with the lethal dose (LD_50_ = 35 mg/kg) previously calculated for rats [25] and this is pertinent when studying the potential that Co^2+^ has for cardiotoxic effects. The rationale for choosing 1 mg/kg as a dose was to demonstrate that even at this low level (much lower than the lethal dose) there are detrimental effects on contractile function of the heart. This is in line with previous work where administration of 5 mg/kg i.p. twice a day caused a 10% reduction in fractional shortening [26]. In examining the echocardiographic parameters of Co^2+^-treated rats in the current study, the key difference between untreated and Co^2+^-treated animals appears to be in the end-diastolic diameter measurement suggesting there may be impaired relaxation of the heart after 28 days of Co^2+^ treatment. This could imply some stiffening or fibrosis of the heart at this stage as a result of effects of Co^2+^ on CFs—the cells responsible for extracellular matrix production. That said, there are no obvious signs of cardiac remodelling even after 28 days (Fig. 3b).

It is important to note that treatment of rats with 1 mg/kg CoCl_2_ resulted in levels of Co^2+^ in whole blood ~ 100-fold higher than untreated animals, 99.01 ± 13.83 and 121 ± 9.88 µg/L on 7 days and 28 days, respectively (Fig. 2). This relates to patients with MoM implants where Co^2+^ levels in serum are more than 100-fold that of physiological levels (≤ 0.29 µg/L) [27]. One case study reported levels of Co^2+^ in patients with MoM implants as 23 µg/L and this corresponded with diastolic dysfunction [28]. It is clear that there is significant patient to patient variability in terms of circulating Co^2+^ from implants. Data from a recent systematic review suggest a mean blood [Co^2+^] of 324 µg/L with some patients showing < 20 µg/L. What is apparent is that higher circulating Co^2+^ results in more marked cardiotoxicity [29]. Importantly, the circulating levels of Co^2+^ in the rats used in this study are within the range of the levels found in patients with hip implants and since we used young (8–10 weeks), healthy adult rats in this study, there are no inherent cardiovascular problems associated with ageing that could impact on our results.

The effects of Co^2+^ on contractile performance in the current study could be a result of direct effects on the contractile cardiac myocytes and/or indirect effects on CFs. Either way, in order for Co^2+^ to exert its effects on the heart there must be efficient cellular uptake mechanisms available to enable rapid entry of the ion to the target cells. We have examined the effects of Co^2+^ treatment on three ion channels/transporters (DMT1, TRPC6 and TRPM7) all of which are expressed in the heart and could potentially transport Co^2+^. Initial data from left ventricular cardiac homogenates prepared from control and Co^2+^-treated rats suggest that chronic (28 day) treatment leads to significant increases in TRP channel (TRPC6 and TRPM7) expression. These channels may be a route by which Co^2+^ enters cardiac cells and may also be a means by which it exerts its cardiotoxic effects.

Based on our in vivo findings suggesting that Co^2+^ causes impaired cardiac relaxation, we examined whether Co^2+^ may specifically target and cause effects on CFs. CoCl_2_ is efficiently taken up into CFs. Increasing the concentration of extracellular Co^2+^ leads to a significantly greater intracellular concentration (Fig. 5a). Importantly, as intracellular Co^2+^ levels rise, CF viability decreases. This becomes particularly obvious at high extracellular Co^2+^ concentrations of > 100 µM (Fig. 5b). CF viability is not affected at concentrations of 25 µM Co^2+^ and below (Fig. 5c). In order to examine whether TRP channel expression is specifically altered in CFs, we treated cells with low levels (10 µM) of CoCl_2_, a concentration we knew would not affect viability but may impact on cellular function and may be more in line with the concentration used for our in vivo work. Intriguingly, and in line with results from homogenised ventricular tissue, we found that TRP channel expression was significantly increased after both 48-h and 72-h treatments with 10 µM Co^2+^ (Fig. 6). The fact that Co^2+^ application to CFs directly results in altered TRP channel expression may suggest involvement of these channels in Co^2+^-mediated cardiac effects. TRPC6 and/or TRPM7 could be a key route of entry for Co^2+^ into CFs. By effecting an up-regulation of these channels, Co^2+^ may be exerting a positive feedback loop enabling more efficient entry to the cells and expediting a cardiotoxic response.

Cardiotoxicity associated with Co^2+^ has not previously been linked with CF function, yet these cells are of paramount importance in maintaining a healthy heart. CFs are quiescent under normal conditions but in response to pathological or toxic stimuli, can become hyper-proliferative and secrete a variety of biochemical mediators that trigger the fibrotic response [30]. The effects that we observe on CF viability following treatment with high (> 100 µM) CoCl_2_ concentrations in the current study do not indicate hyper-proliferation but instead show reduced viability and reduced potential for proliferation, probably as a result of cell damage at higher levels of intracellular Co^2+^. Given these observations, it seems more likely that higher concentrations of Co^2+^ would exert effects on viability rather than proliferation of CFs in the quiescent ‘healthy’ heart. However, in patients with compromised cardiac function, such as pathological remodelling following a myocardial infarction or prolonged hypertension, where the proliferating CF is to the fore, Co^2+^ could have a significant impact both on viability and proliferative potential. We did not assess collagen levels in this study but this is a feature of hyper-proliferation and fibrosis that could be assessed both in whole heart and at a cellular level following treatment with CoCl_2_ to monitor production of extracellular matrix. This would be important in determining any potential effects of CoCl_2_ at a structural level and may link with our observations on altered TRP channel expression following 10 µM CoCl_2_ treatment.

Ca^2+^ signalling plays a crucial role in mediating healthy CF function and TRP channels (TRPM7 in particular) have been shown to play a key role in regulating Ca^2+^ entry to CFs in health and disease [31]. Of particular relevance to the current study, TRP channels, and TRPM7 specifically, have been shown to be up-regulated in patients with cardiovascular pathology. TRPM7 is up-regulated by 3- to fivefold in CFs from patients with atrial fibrillation compared with sinus rhythm patients [32]. It has been suggested that TRPM7-mediated Ca^2+^ signalling could play a pivotal role in the development of fibrosis associated with cardiovascular disease and that TRPM7 could be an effective therapeutic target [14]. In addition to TRPM7, TRPC6 has also been associated with a disease-like phenotype for CFs. Evidence from a genome-wide screen has been presented to suggest a link between up-regulation of TRPC6 and healthy fibroblast to hybrid myofibroblast transformation [33]. Pro-fibrotic ligands such as TGFβ and angiotensin II cause up-regulation and activation of TRPC6 and it has been suggested that inhibitors of TRPC6 could be effective in targeting fibrotic disease. It is pertinent that both TRP channels investigated in the current study and shown to be up-regulated by Co^2+^ treatment, have direct links with a pathological phenotype when their levels and activity are up-regulated. Although we have not assessed TRP channel function in this study, it seems likely that the elevated protein expression following Co^2+^ treatment is associated with increased activity.

It is important to note that Co^2+^ treatment has been used previously as a hypoxia mimetic in cell culture and acts to stabilise and up-regulate hypoxia inducible factor-1α (HIF1α) [34]. TRP channels can be activated by HIF1α and this may occur via direct binding of HIF1α to the TRP channel gene as has been shown for TRPA1 [35]. Whether this mechanism of action may be important for the effects of Co^2+^ in CFs has not yet been examined but it could be a factor in the cardiotoxic response to Co^2+^ exposure. As well as indirect effects on activation of TRP channels mediated via HIF1α, Co^2+^ has also been suggested to directly block certain TRP channels. Co^2+^ can compete with Ca^2+^ and can bind to the ion selectivity filter of TRPV1 so there appears to be a co-entry mechanism and competition for binding sites [36]. Whether Co^2+^ competes with Ca^2+^ entry in TRPC6 and TRPM7 in CFs remains to be established but these experiments will be an important follow-up to the current study in elucidating the mechanism of action of Co^2+^ in these cells and in the heart.

Up-regulation of both TRPC6 and TRPM7 as shown in the whole heart and the isolated CFs from Co^2+^-treated animals could link with the compromised function we see in in vivo. It will be important to link changes in TRP channel function with altered expression and to establish whether inhibition of these Co^2+^-induced effects may restore cardiac contractile function. Investigation of the effects of TRPC6 and TRPM7 inhibition at the level of the CF will show whether there is a link between Co^2+^-induced up-regulation of these channels and abnormal cell function. As such, TRPC6 and TRPM7 targeted interventions could prove therapeutically useful in patients with MoM orthopaedic implants. Improved understanding of how Co^2+^ enters the cells of the heart, as well as how it causes adverse cellular effects, should prove crucial in determining intervention strategies that could limit further damage to the cardiovascular health of these patients.

## References

[1] MHRA (Medicines and Healthcare products Regulatory Agency). (2012). Medical device alert ref. MDA/2012/036. Retrieved June 25, 2012 from https://assets.publishing.service.gov.uk/media/5485abf6ed915d4c10000273/con155767.pdf.

[2] Alexander CS (1972). Cobalt-beer cardiomyopathy: A clinical and pathologic study of twenty eight cases. The American Journal of Medicine.

[3] Nemery B (1992). Survey of cobalt exposure and respiratory health in diamond polishers. The American Review of Respiratory Disease.

[4] Swennen B (1993). Epidemiological survey of workers exposed to cobalt oxides, cobalt salts and cobalt metal. Occupational and Environmental Medicine.

[5] Machado C, Appelbe A, Wood R (2012). Arthroprosthetic cobaltism and cardiomyopathy. Heart, Lung and Circulation.

[6] Mao X, Wong AA, Crawford RW (2011). Cobalt toxicity—an emerging clinical problem in patients with metal-on-metal hip prostheses?. The Medical Journal of Australia.

[7] Prentice JR (2013). Metal-on-metal hip prostheses and systemic health: A cross sectional association study 8 years after implantation. PLoS ONE.

[8] Afolaranmi GA (2012). Distribution of metal released from cobalt-chromium alloy orthopaedic wear particles implanted into air pouches in mice. Journal of Biomedical Materials Research Part A.

[9] Afolaranmi GA, Grant MH (2013). The effect of ascorbic acid on the distribution of soluble Cr and Co ions in the blood and organs of rats. Journal of Applied Toxicology.

[10] Zheng J (2013). Molecular mechanisms of TRP channels. Comprehensive Physiology.

[11] Monteilh-Zoller MK (2003). TRPM7 provides an ion channel mechanism for cellular entry of trace ion metals. The Journal of General Physiology.

[12] Topala CN (2007). Molecular determinants of permeation through the cation channel TRPC6. Cell Calcium.

[13] Rowell J, Koitabashi N, Kass DA (2010). TRP-ing up heart and vessels: Canonical transient receptor potential channels and cardiovascular disease. Journal of Cardiovascular Translational Research.

[14] Yue Z, Xie J, Yu AS, Stock J, Du J, Yue L (2015). Role of TRP channels in the cardiovascular system. American Journal of Physiology-Heart and Circulatory Physiology.

[15] Ke Y, Chen YY, Chang YZ, Duan XL, Ho KP, Jiang DH (2003). Post-transcriptional expression of DMT1 in the heart of rat. Journal of Cellular Physiology.

[16] Skorringe T, Burkhart A, Johnsen KB, Moos T (2015). DMT1 in the brain: Implications for a role in iron transport at the blood brain barrier and neuronal and glial pathology. Frontiers in Molecular Neuroscience.

[17] Howitt J, Putz U, Lackovic J, Doan A, Dorstyn L, Cheng H (2009). DMT1 regulation by Ndfip1 prevents metal toxicity in human neurons. Proceedings of the National Academy of Sciences USA.

[18] Camelliti P, Borg TK, Kohl P (2005). Structural and functional characterisation of cardiac fibroblasts. Cardiovascular Research.

[19] Mooney L, Skinner M, Coker SJ, Currie S (2015). Effects of acute and chronic sunitinib treatment on cardiac function and CaMKII. British Journal of Pharmacology.

[20] Martin TP, Lawan A, Robinson E, Grieve DJ, Plevin RJ, Paul A, Currie S (2014). Adult cardiac fibroblast proliferation is modulated by calcium/calmodulin dependent protein kinase II in normal and hypertrophied hearts. Pflügers Archiv.

[21] Lowry OH, Rosebrough NJ, Farr AL, Randall RJ (1951). Protein measurement with the Folin phenol reagent. Journal of Biological Chemistry.

[22] Tallquist MD, Molkentin JD (2017). Redefining the identity of cardiac fibroblasts. Nature Reviews Cardiology.

[23] Galbraith RA, Kappas A (1991). Cobalt protoporphyrin regulates body weight in beagle dogs: Induction of weight loss in normal animals of stable adult weight. Pharmacology.

[24] Galbraith RA, Kappas A (1989). Regulation of food intake and body weight by cobalt porphyrins in animals. Proceedings of the National Academy of Sciences USA.

[25] Van Liew HD, Chen PY (1972). Cardiorespiratory functions during histotoxic hypoxia caused by cobalt. Journal of Applied Physiology.

[26] Murakoshi N (2000). Impairment of cardiac energy metabolism in vivo causes hemodynamic abnormality and increases cardiac expression of preproendothelin-1 mRNA. Journal of cardiovascular pharmacology.

[27] De Smet K, De Haan R, Calistri A, Campbell PA, Ebramzadeh E (2008). Metal ion measurement as a diagnostic tool to identify problems with metal on metal resurfacing. The Journal of Bone and Joint Surgery.

[28] McLaughlin, J., & Castrodale, L. (2010). Cobalt toxicity in two hip replacement patients. *State of Alaska Epidemiology Bulletin*, 14.

[29] Gessner BD, Steck T, Woelbar E, Tower SS (2015). A systematic review of systemic cobaltism after wear or corrosion of chrome-cobalt hip implants. Journal of Patient Safety.

[30] Swynghedauw B (1999). Molecular mechanisms of myocardial remodelling. Physiological Reviews.

[31] Yue Z, Zhang Y, Jiang J, Yue L (2013). Transient receptor potential (TRP) channels and cardiac fibrosis. Current Topics in Medicinal Chemistry.

[32] Du J, Xie J, Zhang Z, Tsujikawa H, Fusco D (2010). TRPM7-mediated Ca^2+^ signals confer fibrogenesis in human atrial fibrillation. Circulation Research.

[33] Davis J, Burr AR, Davis GF, Birnbaumer L, Molkentin JD (2012). A TRPC6-dependent pathway for myofibroblast transdifferentiation and wound healing in vivo. Developmental Cell.

[34] Dai Z, Gao J, Ma X, Yan K, Liu X (2012). Up-regulation of HIF-1α by cobalt chloride correlates with proliferation and apoptosis in PC-2 cells. Journal of Experimental & Clinical Cancer Research.

[35] Hatano N, Itoh Y, Suzuki H, Muraki Y, Hayashi H, Onozaki K (2012). Hypoxia-inducible factor 1α switches on transient receptor potential ankyrin repeat 1 (TRPA1) gene expression via a hypoxia response element-like motif to modulate cytokine release. Journal of Biological Chemistry.

[36] Pecze L, Winter Z, Josvay K, Otvos F, Kolozsi C, Vizler C (2013). Divalent heavy metal cations block the TRPV1 Ca^2+^channel. Biological Trace Element Research.

